# Eigenbehaviour as an Indicator of Cognitive Abilities

**DOI:** 10.3390/s22072769

**Published:** 2022-04-04

**Authors:** Angela A. Botros, Narayan Schuetz, Christina Röcke, Robert Weibel, Mike Martin, René M. Müri, Tobias Nef

**Affiliations:** 1 Gerontechnology and Rehabilitation Group, ARTORG Center for Biomedical Engineering Research, University of Bern, 3008 Bern, Switzerland; angela.botros@unibe.ch (A.A.B.); narayan.schuetz@unibe.ch (N.S.); rene.mueri@insel.ch (R.M.M.); 2 University Research Priority Program (URPP) “Dynamics of Healthy Aging”, University of Zurich, Andreasstrasse 15, 8050 Zurich, Switzerland; christina.roecke@uzh.ch (C.R.); m.martin@psychologie.uzh.ch (M.M.); 3 Department of Geography, University of Zurich, Winterthurerstrasse 190, 8057 Zurich, Switzerland; robert.weibel@geo.uzh.ch; 4 Department of Psychology, University of Zurich, Binzmühlestrasse 14, 8050 Zurich, Switzerland; 5 Department of Neurology, Inselspital, University Hospital of Bern, University of Bern, 3010 Bern, Switzerland

**Keywords:** pervasive computing, home monitoring, cognitive ability, dementia, mild cognitive impairment, digital biomarkers, digital measures

## Abstract

With growing use of machine learning algorithms and big data in health applications, digital measures, such as digital biomarkers, have become highly relevant in digital health. In this paper, we focus on one important use case, the long-term continuous monitoring of cognitive ability in older adults. Cognitive ability is a factor both for long-term monitoring of people living alone as well as a relevant outcome in clinical studies. In this work, we propose a new potential digital biomarker for cognitive abilities based on location eigenbehaviour obtained from contactless ambient sensors. Indoor location information obtained from passive infrared sensors is used to build a location matrix covering several weeks of measurement. Based on the eigenvectors of this matrix, the reconstruction error is calculated for various numbers of used eigenvectors. The reconstruction error in turn is used to predict cognitive ability scores collected at baseline, using linear regression. Additionally, classification of normal versus pathological cognition level is performed using a support-vector machine. Prediction performance is strong for high levels of cognitive ability but grows weaker for low levels of cognitive ability. Classification into normal and older adults with mild cognitive impairment, using age and the reconstruction error, shows high discriminative performance with an ROC AUC of 0.94. This is an improvement of 0.08 as compared with a classification with age only. Due to the unobtrusive method of measurement, this potential digital biomarker of cognitive ability can be obtained entirely unobtrusively—it does not impose any patient burden. In conclusion, the usage of the reconstruction error is a strong potential digital biomarker for binary classification and, to a lesser extent, for more detailed prediction of inter-individual differences in cognition.

## 1. Introduction

Assessing cognition in older adults is a highly relevant task. This holds true for both conducting clinical trials [1,2,3] as well as in aging-focused home-monitoring applications [4,5]. In both cases, continuous long-term monitoring can complement the existing point-in-time, snapshot examinations commonly conducted in clinical practice [6].

For the measurement of cognitive abilities, there are a numerous different tests. The Mini-Mental State Exam (MMSE) [7] is an assessment to test for mild cognitive impairment (MCI) [8], Alzheimer’s disease (AD) [9] and other cognitive issues. This test has a maximum cognitive ability score of 30. A cutoff is used to distinguish between normal cognitive ability and people with dementia. A generally accepted cutoff score for the MMSE is 24 [7,10], but a more differentiated interpretation is suggested with adaptive cutoffs depending of the individuals’ age and education [11]. Another test to detect cognitive impairment is the Montreal Cognitive Assessment (MoCA) [12]. This test has a maximum score of 30, too. The cutoff to distinguish between people with normal cognitive ability and people with MCI or AD is set at 26 [13], but this value has been up for debate [12]. Other evaluations usually cover a selection of individual tasks, such as the Halstead–Reitan neuropsychological test battery [14]. Besides these non-invasive assessments, there are also more expensive and invasive methods for cognitive assessment. Magnet resonance tomography (MRI), a rather expensive measurement technology, has been shown to be an effective method to assess MCI and risk of AD [15]. Another method is the invasive lumbar puncture, which is used for the detection of biomarkers that can be early indicators of AD or PD [16,17]. However, due to its invasiveness, the safety of this method has been questioned in the past [18,19].

The idea that certain medical conditions can affect daily behaviour has been studied by Paraschiv-Ionescu et al., who monitored physical activities of chronic pain patients over multiple days, and compared the data with those of healthy, pain-free individuals. They specifically noted the change in complexity of physical activities between chronic-pain patients and pain-free patients, with the latter showing more complex behaviour [20].

Pervasive computing is a promising approach towards long-term health monitoring in older adults. It can potentially provide safety and security for independently living individuals, while still maintaining simplicity and—depending on the chosen technology—privacy. Several studies have provided evidence for both acceptance by the target population as well as utility of such systems [21,22,23,24,25,26].

Although it seems possible to use wearable devices to obtain information about cognitive abilities in older adults [23,27,28], most successful research in this regard has been conducted using contactless, unobtrusive sensing technologies [29]. This is especially true for long-term monitoring beyond a few months. The predominant reason for this is likely that with declining cognitive abilities, it may prove increasingly difficult for impaired older adults to maintain devices (for instance remembering to wear and correctly charge them). As such, a majority of research regarding long-term monitoring of cognitive abilities in older adults has focused on technologies, commonly including: passive infrared (PIR) motion sensors and door contact sensors [21,22,25,29,30], amongst others.

The necessity for continuous monitoring of parameters that determine cognitive abilities has been highlighted in [31]. They provide an extensive overview over other studies, ranging from short-term to mid-length over to long-term measurements, and describe how those parameters might fluctuate over time. This is an important aspect for the measurements of cognitive abilities, as these fluctuations could occur naturally, and a good average value would be the best measurement outcome. Alternatively, when these fluctuations are an indicator of a problem in itself, then they should be captured by repeated measurements and characterized as any other disease symptom would.

In recent years, there have been approaches using machine learning techniques for the assessment of cognitive abilities. In a study involving 79 older adults with and without dementia, a set of scripted movements was performed by the participants in a natural-like environment [32]. The participants movements were recorded using contactless ambient sensors. An extensive list of extracted features was extracted including the duration to perform the scripted activities and the participants age. Multiple different algorithms such as naive Bayes, decision tree, sequential minimal optimization and neural networks were tested for their ability to distinguish between the cognitively health and those with dementia.

In a very recent study [33], similar features as used in [32] have been used on the Cognitive Assessment Activity (Kyoto) dataset [34]. In this dataset, scripted activities in a natural-like test environment were recorded with a total of 400 participants. The contactless ambient sensors recording the movements are similar to those used in [32].

In a large study, including 97 participants and taking measurements over a total span of three years, the usability of gait speed as an indicator for MCI was studied [35]. In this study, contactless and non-obtrusive PIR technology was used to measure the movement throughout the apartments and infer the gait speed. Eventually, 24-week-long measurement segments were used in the classification of the cognitive abilities. Both support-vector machines and random forests were tested for their classification performances.

An extensive analysis of machine learning techniques for the activity recognition and detection of abnormal behaviour was performed by Arifoglu et al. [36]. Using a dataset by Van Kasteren et al. [37], they tested a variety of recurrent neural networks (RNN), namely a vanilla RNN, a GRU RNN and an LSTM for their usability to recognise activity and detect abnormal behaviour.

Unlike the two studies by Dawadi et al. [32] and Javed et al. [33], which rely on the detection and measurement of specific activities, in [38], less activity-focused data was used for the assessment of depression. The inability of the ambient sensors used to distinguish between specific abilities is countered by the usage of long-term data spanning multiple months up to a year and covering 13 participants.

Research from our group has shown that the daily routine of participants diagnosed with MCI and AD is far less regular and more chaotic than the daily behaviour of age-matched healthy individuals [25]. This finding is supported by a larger body of research, showing that increased variability in physical activity [21,30], gait-speed [22,29,30,39] and other factors of daily behaviour [27,28] are often associated with mild cognitive impairment (MCI).

One of the major problems of the previously mentioned research from our group [25] was validating ADL recognition performance in people with cognitive impairments—as self reporting is not reliable in this case and more intrusive ways of validation, such as video recordings, would be ethically questionable at best. Thus, in this article, we evaluate a purely location-based approach to estimate regularity of behaviour. For this, we use contacless, unobtrusive sensors consisting of PIR motion and door contact sensors. For the analysis of the behaviour regularity, we suggest a method based on eigen-decomposition of behavioural matrices, a method introduced by Eagle et al. [40]. They used the approximate localisation data obtained from cell phones of 100 student participants. An eigenvalue decomposition on this data provided insight into the students’ behaviour, organisational group and circle of friends. The idea to use principal component analysis to analyse the underlying structure of data is not new, it was notably used in [41], where principal component analysis is conducted to represent faces [20].

The novelty of this paper is the introduction of a method for the assessment of cognitive ability based on unobtrusive contactless measurements at home. This paper discusses three relevant aspects:

- First, a new method for location movement patterns is introduced.
- Second, the usage of this method for assessment of cognitive ability is demonstrated. This includes a discussion of the necessary hyper parameters and their validity.
- Finally, the usability of this method for the assessment of cognitive ability is demonstrated on the data of 48 participants, all above the age of 65, i.e., retirement age.

## 2. Materials and Methods

### 2.1. Participants

The data presented in this study stems from two studies, the StrongAge Cohort Study and the MOASIS MobiPro Study. Both studies were conducted based on the principles declared in the Declaration of Helsinki and approved by the University of Zurich Ethics Committee and the Ethics Committee of the Canton of Bern, respectively. All participants signed and handed in an informed consent form before study participation.

The StrongAge Cohort Study is a home-monitoring study, where community-dwelling seniors (inclusion criterion ≥80 years) were equipped with pervasive computing systems for approximately one year [21]. The recruitment aimed to represent a naturalistic sample of alone-living older adults in central Switzerland, irrespective of their cognitive ability.

The MOASIS MobiPro Study is a home-monitoring study, where community dwelling seniors (inclusion criterion ≥65 years) were equipped with pervasive computing systems for approximately four weeks [42]. The aim of the study was to assess mobility, physical and social acitivity patterns in relation to health and well-being in healthy older adults. For the current analysis, only alone-living participants of the MOASIS MobiPro Study were selected.

### 2.2. Data Collection

In this study, passive infrared (PIR) sensors (DomoSafety SA, Lausanne, Switzerland) were used to monitor the participants in their respective homes. These commercially available sensors have been validated in multiple studies [21,43]. The PIR sensors were placed in order to cover the relevant living spaces: bedroom, kitchen, bathroom, living room and entrance area. These sensors recorded presence or absence of movement with a frequency of 0.5 Hz. In addition to the PIR sensors, door sensors were placed on the entrance door and the fridge to assess time outside of home as well as kitchen usage. The sensors were installed in the participants’ homes at the beginning of the study and disassembled again at the end. At the beginning of the respective studies, participants’ cognitive ability was assessed with a diverse battery of tests. The MoCA score was used in this project as a measure of cognitive ability. The sensor-based activity and mobility monitoring in the StrongAge Cohort Study was conducted for up to a year. The monitoring in the MOASIS study was conducted over a span of four weeks. To avoid any biases, the data from the StrongAge Cohort Study was sub-sampled. Both time points of measurement as well as number of days distributions were matched. The obtained data is not publicly available due to local Swiss data regulations.

### 2.3. Behaviour Matrix and Eigendecomposition

**Figure 1:**
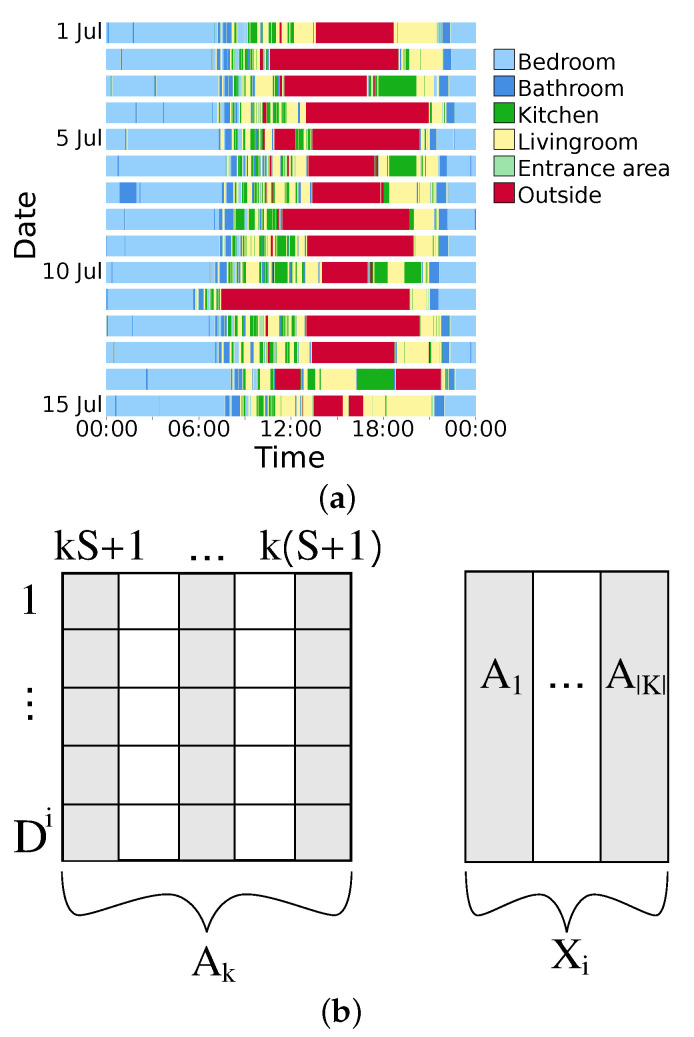
In (**a**) a representation of multiple days is shown, with different room locations colour coded. In (**b**), the structure of matrix $X^{i}$ is explained. Along the columns, sub-matrices $A_{k}$ are stacked. Every sub-matrix $A_{k}$ contains the presence percentages of location *k*. Its width is *S* and its height is $D^{i}$, the total measurement days of person *i*. The total matrix $X^{i}$ consists of the horizontally stacked matrices $A_{k}$.

The PIR sensor data consists of time and duration of activation for all sensors. Based on this, the location of the people in their apartment throughout each day was obtained [21]. The set of locations is K={bedroom,bathroom,livingroom,kitchen,entrance,outside}.A visual representation of the locations as estimated by the sensors is given in Figure 1a. For the eigenbehaviour, every day of data is subdivided into *S* time windows, each of length $\Delta t=\frac{24h}{S}$. A different number of *S* were assessed, with S:={24,48,96,144,288} resulting in window lengths ranging from 5 min (S=288) up to 1 h (S=24). For every time window, the percentage of presence in every room was calculated.

For every person *i*, a location matrix $X^{i}$ was computed. Every row is a day of measurement, with a total height of $D^{i}$-measurement days for person *i*. In the columns, the percentages of presence for every time window and location are given. The locations are stacked horizontally, i.e., the first *S* columns represent the first location, and the columns {(k−1)·S,(k−1)·S+1,⋯,k·S−1} represent the time windows of location *K* for k=1⋯|K|. The resulting $X^{i}$ is a $|D^{i}\left|\times S\cdot |K\right|$ matrix. This is also shown in Figure 1b.

In every individual cell $X^{i}\left[d_{j},k\cdot S+n\right]$, the percentage of presence in the corresponding location *K* on day $d_{j}$ in the time window $\left[\frac{24h}{\Delta t}n,\frac{24h}{\Delta t}\left(n+1\right)\right]$ is given. This fragmentation was the same for all participants.

The j−th row of $X^{i}$ is $\Gamma_{j}^{i}$ and represents exactly one day, or one point in an (S·|K|)-dimensional space. The average location vector of person *i* is $\Psi^{i}=\frac{1}{D^{i}}\sum_{d=1}^{D^{i}}\Gamma_{j}^{i}$. The deviation of an individual day from the average day is $\Phi_{j}^{i}=\Gamma_{j}^{i}-\Psi^{i}$. The location deviation matrix is ${\hat{X}}^{i}=\left[\Phi_{1}^{i},\cdots ,\Phi_{D^{i}}^{i}\right]\in R^{D^{i}\times \left(S\cdot |K|\right)}$. To analyse the different behaviours for every person *i*, principal component analysis is performed on the collection of vectors $\Phi_{j}^{i}$. The covariance matrix $C^{i}$ of person *i* is based on this set:

(1) $$C^{i}={\hat{X}}^{i}{\left({\hat{X}}^{i}\right)}^{T},\;\;s.t.\;\;c^{i}\left[n,m\right]=\sum_{d=1}^{D^{i}}\Phi_{d}^{i}\left[n\right]\Phi_{d}^{i}\left[m\right]$$

From the covariance matrix $C^{i}$ of person *i*, the eigenvectors $v_{l}^{i}$ and eigenvalues $\lambda_{l}^{i}\left(C^{i}\right)$ can be computed. They represent the principal components of the deviation vectors $\Phi_{j}^{i}$.

(2) $$\begin{array}{cc} C^{i}= & U^{i}\cdot \Lambda^{i}\cdot \left(V^{i}\right)T \end{array}$$

(3) $$\begin{array}{cc} {\tilde{C}}^{i}= & C\cdot U_{k}^{i}{\left(U_{k}^{i}\right)}^{T} \end{array}$$

(4) $$\begin{array}{cc} e_{k}^{i}= & \left|\right|C^{i}-{\tilde{C}}^{i}{\left|\right|}_{F}^{2} \end{array}$$

To compute the *k*-th reconstruction error $e_{k}^{i}$ of person *i*, a singular value decomposition is performed on the covariance matrix $C^{i}$, as in Equation (2). The matrices $U^{i}$ and $V^{i}$ are unitary matrices, while the square diagonal matrix $\Lambda^{i}$ contains the singular values of $C^{i}$. To only consider the *k* largest singular values and dismiss the smaller values, only the first *k* columns of the matrix $U^{i}$ are used, resulting in a smaller matrix $U_{k}^{i}$. The covariance matrix is reconstructed using only the entries corresponding to the *k* largest singular values as in (3). The reconstruction error is the difference of the original covariance matrix $C^{i}$ and the newly reconstructed covariance matrix $C_{k}^{i}$ (4).

The number of singular values *k* that are used for the reconstruction determines the degree to which the resulting reconstructed matrix deviates from the original one. The difference between the reconstructed matrix and the original matrix is the reconstruction error. We refer to the reconstruction error obtained from using only the first eigenvector as the first reconstruction error, and the reconstruction error obtained from using the first *n* eigenvectors as the *n*th reconstruction error. As described in [40], the first singular values correspond to regular behaviour, while the smaller singular values usually correspond to more irregular, noisier aspects of the original matrix. As observed in [25], people with lower cognitive abilities tend to loose their regular daily pattern, and show a more irregular, chaotic behaviour. This would lead to a reduced prominence of the effect of the first few singular values, and more importance on the last values. Thus, by cutting off the later singular values, the reconstruction error would increase for people, where more chaotic behaviour is displayed.

### 2.4. Prediction and Classification of Cognitive Ability

In order to assess the influence of age on the prediction of the cognition score, the partial correlation of the cognitive ability score and the reconstruction error were computed, with age as the confounding variable.

The cognition score was predicted with a linear regression, using the reconstruction error and age as features. As a baseline, a regression was trained using only age and no reconstruction error. Cross-validation was used for evaluation due to the small sample size. The root-mean-square deviation RMSD $=\frac{1}{N}\sum_{i}\left({\left(y_{i}-{\hat{y}}_{i}\right)}^{2}\right)$ of the predicted score ${\hat{y}}_{i}$ and true score $y_{i}$ over all *N*-measured individuals was employed as the accuracy measure.

Besides the cognition prediction, a more general classification was performed, where the participants were divided into two groups; those with a score at or above 26, and those with a score below 26. A support vector machine (SVM) with a sigmoid kernel was used for the classification. Class weights were balanced. As a reference, a score at or above 26 is considered normal, while a score below this value indicates mild to severe cognitive impairment [12]. Three-fold cross-validation was used, where the split was conducted with two-thirds of the data (=32 samples) in the training set and one-third (=16 samples) in the validation set, with the two sets stratified. The receiver operating characteristic (ROC) and its area under the curve (AUC) were calculated for all cross-validation folds. The mean ROC and the confidence interval (± std) were evaluated for final assessment. As a comparison, the same classification is also performed using age as the only classification feature.

The optimization parameters were the number of eigenvectors used for the reconstruction and the size of the time window *S*. For both prediction and classification, a simultaneous grid search was performed in a leave-one-out evaluation for parameter optimization.

All preprocessing and calculations were performed using the Python programming language, version 3.6.9 (Python Software Foundation). Correlations and significances thereof were calculated using the Python package scipy.stats, version 1.3.1. Figures and graphical illustrations were created using the above-mentioned Python programming language, as well as Inkscape, version 1.0.

## 3. Results

**Figure 2:**
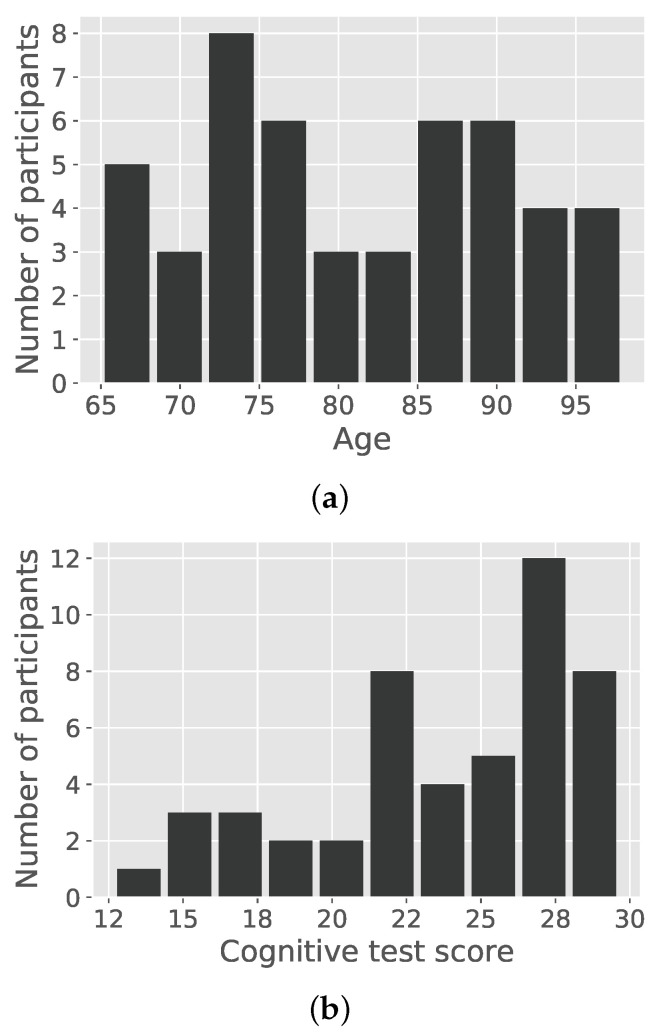
In (**a**), the age distribution of the participants is depicted. It closely follows a uniform distribution. In (**b**), the score of the cognitive test is depicted.

In this study, data from a total of 48 people were evaluated (38 women and 13 men). Of those people, 20 were from the MOASIS MobiPro Study and 18 from the StrongAge Cohort Study. The participants were all above retirement age, with mean age of 81.08 (SD 9.73) and mean cognition score of 23.88 (SD 4.54). The age distribution is close to being uniformly distributed between the ages of 65 and 98. The Kolmogorov–Smirnov statistic, when comparing the age values to the uniform distribution, is D=0.064 with a *p*-value of 0.989. Both age and cognition distributions are shown in Figure 2a,b. The people were monitored on average for 30.6 days (SD 3.6)—excluding start and end day of measurements.

The partial correlations of the three parameters age, cognition and first reconstruction error were computed. The results are presented in Table 1. There was a slight positive correlation between age and the reconstruction error at ρ=0.27, which was not significant. Age has a noticeable negative correlation with the cognition score, which was significant at p<0.01. The reconstruction error and the score had the strongest correlation, at ρ=−0.42. This correlation was highly significant, at p<0.005.

**Figure 3:**
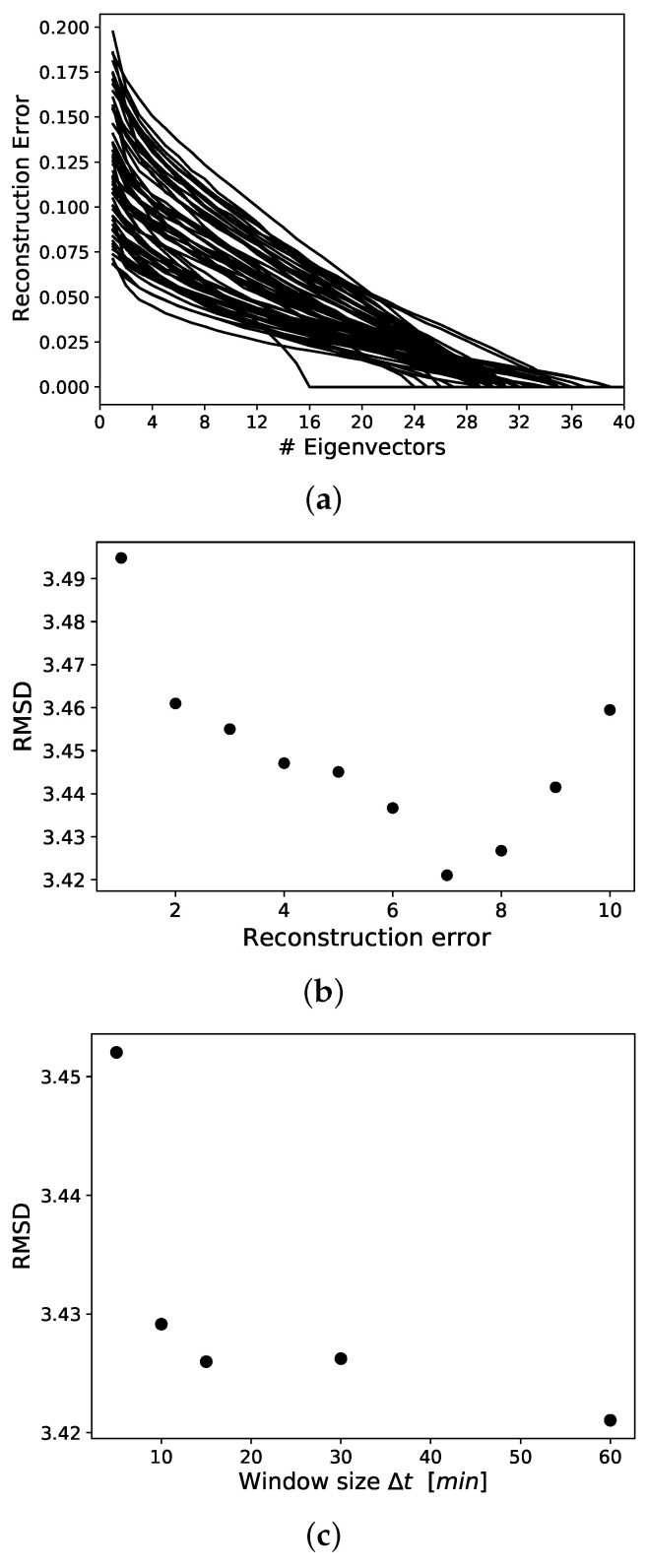
In (**a**), the normalized reconstruction error for increasing numbers of used eigenvectors is depicted. In (**b**), the RMSD of a linear regression for different reconstruction errors is shown. In (**c**), the optimal window size Δt is evaluated. The lowest RMSD is obtained at Δt = 60 min ↔S=24.

Based on the behaviour matrix, the reconstruction errors were computed. They decreased for increasing numbers of included eigenvectors up until their vanishing point when $|D_{i}|$ eigenvectors were used for the reconstruction. This is depicted in Figure 3a, where the segmentation was set at S=24 resulting in one-hour-long time segments. For other segmentations, the structure of the reconstruction errors looked similar.

First, the results of the parameter optimization are presented. As a baseline, the RMSD of the baseline was computed—the linear regression which was based only on age as a feature and no reconstruction error. This resulted in an RMSD of 3.74, higher than any of the regressions including the reconstruction errors. The RMSD for the cognitive ability prediction was computed for all reconstruction errors and is shown in Figure 3b up to the 10th reconstruction error. The best performance, i.e., the lowest RMSD, was obtained when using the 7th reconstruction error. This is shown in Figure 3b, where the segmentation is set at S=24. For all other S={48,96,144,288}, similar results were obtained, with the 7th reconstruction error being the best choice for the prediction.

In Figure 3c, the RMSD is depicted for S={24,48,96,144,288}, equivalent to window sizes of Δt = { 60 min, 30 min, 15 min, 10 min, 5 min}. The 7th reconstruction error was used in this figure. For S=288, the RMSD is substantially higher than for the other chosen window sizes. The results for S={24,48,96} are very close together, but the lowest RMSD is obtained for S=24, i.e., Δt = 60 min.

**Figure 4:**
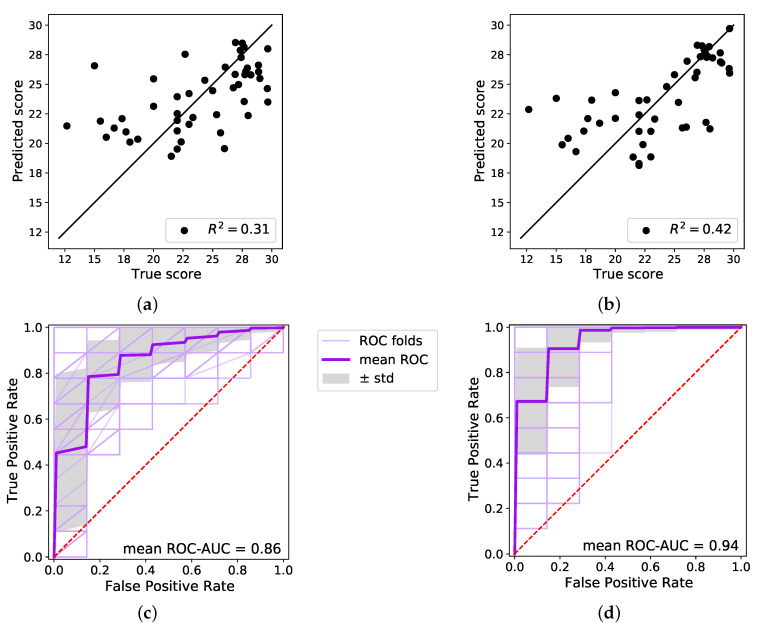

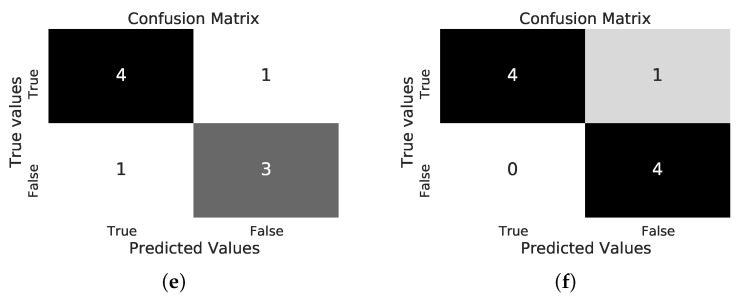
On the left, in (**a**,**c**,**e**), the prediction and classification results are only based on age. On the right, in (**b**,**d**,**f**), the prediction and classification results are based on age and the reconstruction error. In (**a**,**b**), linear regression results are shown. In (**c**,**d**), the classification results are shown. The regression in (**b**) and the classification in (**d**) are based on the optimal window size Δt=60 min and the optimal 7th reconstruction error. In (**c**,**d**), the purple line is the mean ROC of the classification. The thin lines indicate all individual runs. In (**e**,**f**), confusion matrices for the classification of the test set are depicted. The test set contains nine samples, five of people with MCI and four of people with normal cognitive abilities, reflecting the overall distribution.

The linear regression model was evaluated in a leave-one-out cross-evaluation. As a baseline, a coefficient of determination $R^{2}=0.31$ was obtained for the prediction, using only age as input feature. This is shown in Figure 4a, where the black solid line indicates optimal performance. When including the reconstruction error, the coefficient of determination increases to $R^{2}=0.42$ as is depicted in Figure 4b. Overall, the predictions were more concise for higher cognition scores, while for lower scores the prediction became worse and spread out. The RMSD of the linear regression with window size Δt=60 min using the 7th reconstruction error was RMSD = 3.42.

In the classification task, the SVD was used with a sigmoid kernel. The coef0 was 0.05 and the classes were balanced due to there being slightly more samples in the MCI class. In the classification task, the effect of the reconstruction error was tested as well. The classification with only age as a feature resulted in an AUC of the mean ROC of AUC =0.86. The classification with both the reconstruction error and age as features resulted in an AUC of the mean ROC of AUC =0.94. Thus, the inclusion of the reconstruction error led to an increase of 0.08 points. For both classifications, the variation was fairly high between the individual runs. Both ROCs are shown in Figure 4c,d, including the standard deviation of the multiple ROC runs.

For the classification task, the confusion matrix was computed for the test set, both for the age-only classification and the one including the reconstruction error. This is shown in Figure 4e,f. It is important to note here that the test set is rather small, with only nine sample points.

## 4. Discussion

In this work, we showed how PIR-sensor-based location information could be used to gain insights into the cognitive ability of older individuals monitored over one month. Based on the available location information, an eigendecomposition was made, which is sensitive to the regularity in the behaviour patterns. The more predictably and regularly the participants moved around in their apartment, the fewer eigenvectors were required to reconstruct their behaviour. Lower levels of cognitive ability have been found to be associated with a loss of routine [25]. A loss of routine, or more erratic behaviour, is harder to map onto fewer eigenvectors. Thus, the reconstruction error is larger than compared with regular and predictable behaviour.

In our evaluation, we looked at two usages of the reconstruction error in order to predict the cognitive ability. First, we conducted a prediction of the cognition score based on the reconstruction error. Second, we classified the results into a healthy group versus a group with mild to severe cognitive impairment.

For both the prediction as well as the classification of the score, two parameters were optimized: the window-length *S* and the choice of reconstruction error. The error for the time window of 60 min is the smallest, but only by a small margin as compared with the other time windows of 10 min up to 30 min. For the time window of 5 min (S=288), the error increases substantially. It is likely that in our everyday routine, there is a lower boundary on our time precision. A boundary below which it is no longer possible to distinguish between routine behaviour and erratic or chaotic behaviour. For example, if we set an alarm for getting up in the morning, the time we actually get up might still differ by a few minutes, influenced by our mood, our sleep quality or something else. By checking different window lengths, it seems this time window is between five and ten minutes. This would mean that in our routine behaviour, we tend to be exact down to a lower resolution of about 10 min. Another consideration is the computational time. The computation of the eigenvalues is considerably more demanding for a |K|·S×|K|·S=5·144×5·144 matrix than for a 5·24×5·24 matrix, and thus the choice of time resolution should take the computational resources into consideration.

The other parameter that was optimized was the choice of reconstruction error. In the work of Eagle et al. [40], the number of eigenvectors needed to achieve a certain level of reconstruction was used to distinguish between different population groups. In a similar matter, we looked for the best number of eigenvectors needed for the reconstruction error being able to best distinguish interindividual differences in cognition. The most common everyday structural routines are covered by the first few eigenvectors. Due to repeating structures of different time frames—hourly, daily and weekly. Too few eigenvectors would not be able to capture all of this behaviour. On the other hand, when adding too many eigenvectors for the reconstruction, they no longer explain predictable behaviour but actual behavioural noise. This behavioural noise is probably best explained by our own timely inaccuracies as discussed in the previous paragraph as well as disturbances from the outside world. Interestingly, the optimal number of eigenvectors found in our analysis was always seven. While this could be coincidental, it could just as well hint to the periodicity of weekly behaviour patterns. A similar discovery was made by [40], where certain eigenvectors cover specific behavioural aspects, such as weekends or breaks.

For the subgroup classification, the data was split into two groups: the group with a cognition score at or above 26, and the group with a cognitive ability score below 26. The rationale behind this split was the close relationship to the cognitive ability. A cognitive ability score at or above 26 is commonly considered to coincide with normal cognitive ability, whereas a cognitive ability below 26 is connected to MCI or AD [12]. The AUC from the classification of the age is slightly higher than other literature has reported, especially given that other work with much more individual features achieve performance around 0.7–0.8 [27]. This could be explained by the comparably small sample and the wide age distribution. Nevertheless, adding the reconstruction error to the classification has improved the AUC by 0.06. There is still room for variance, and the evaluations would best be repeated with larger data sets. Nevertheless, an improvement of 0.06 is still decent and shows that behaviour regularity captures aspects of cognition beyond just age alone. While the confusion matrix is looking rather promising, it is important to note here that with only nine sample points in the test set this could just as well be a lucky outlier. Only repetitions of these measurements with more people would confirm this rather strong connection in a significant manner.

Due to the small sample size, further splitting of the data into a third group with cognitive ability below 17, as suggested for AD [12], could not reasonably be performed.

Our method shows good prediction behaviour for higher cognition scores but worse performance when the actual cognitive ability score is below 20. On the one hand, we have fewer data points in that area to train a model with, which could explain this lower performance. On the other hand, there are numerous different reasons for low cognitive ability score; reduced language comprehension, working memory, concentration and attention are some of the abilities needed to reach high scores. As the evaluation does not differentiate between the different causes for lower scores, their effect on the movement behaviour is variable. This is not taken into consideration in this evaluation. As with the classification task, the prediction improved by increasing the $R^{2}$ value from 0.31 to 0.42 when adding the reconstruction error. In a future study, more thorough evaluation of the participants and classification of their cognitive ability could improve on these results.

While not all causes for a lower cognitive ability score might lead to a change in movement patterns, there might also be factors present causing changing patterns which are not represented through cognitive abilities. An example was given by Paraschiv-Ionescu et al. in their study covering chronic pain and its effect on physical activity patterns [20]. Furthermore, there is a reasonable chance that some causes for a lower cognitive ability score might even favour the regularity of patterns and increase them. These uncertainties indicate the limitations of this method.

In [32], multiple machine learning algorithms have been tested for their ability to distinguish between cognitively healthy people and people with dementia. While similar sensor technology was used for the measurements, only scripted activities were measured. They have reported similar AUC for their classification task, ranging between 0.80 (decision tree) up to 0.86 (naive Bayes and neural networks). By having more participants and more samples per participants, they were able to more effectively use techniques that are dependent on larger amounts of training data. Their work was further continued in [44,45], where larger studies with more participants were conducted. More in-depth assessment of different algorithms were performed, with more robust results. They have obtained similar AUC values as in our study, varying for different activities. In contrast to our approach, they observed individual activities and tasks, thus measurements and results were obtained faster.

The results obtained by [33] outperform those by [32]. While more participant data is available, there are also more different algorithms tested for the classification. Their best algorithm is able to perform with an AUC of 0.94. However, as in the previously mentioned work, the sensors were monitoring specific activities. While this approach is faster, measurements are obtained more quickly than with our 4-week measurement approach; there is a necessity for better sensors, able to distinguish between different activities and measure the target activities. In contrast, there are studies with a large number of participants measured over shorter periods of time, and there are studies with fewer participants recorded over much longer periods [38]. This trade-off between time and participant allows for different approaches. Using fewer participants but compensating with longer measurement periods might lead to less precise results, but can be beneficial for the perceived privacy of the sensors, and thus increase acceptance by the participants.

In the study by Akl et al., both large numbers of participants and long measurement times have been utilised to achieve a very high classification accuracy for MCI with an AUC of 0.97 [35]. The SVM approach delivered the best performance, but long measurement segments (24 weeks) were strictly necessary to achieve these result. Shorter segments performed significantly worse: their first evaluations using 4 weeks of measurements only delivered an AUC of 0.81. So, while their approach outperforms ours, the necessity of six times as much data might be a problem for evaluation.

While to work by Arifoglu et al. [36] differs in its main target, they focus on the detection of abnormal behaviour and not specifically MCI, they evaluate their dataset on an impressive range of machine learning algorithms. Set into context, the dataset is more comparable with the ones used by Dawadi et al. [32] and Javed et al. [33]. So, the inclusion of more sophisticated machine learning algorithms, specifically those exploiting time-dependent data such as LSTMs and other RNN, is an important next step for the automatic detection and classification of cognitive abilities.

In this study, the complete measurement of four weeks was treated as one individual sample. Thus, the measurements of the 48 participants resulted in only 48 sample points, even though the collected data more closely covers 700 h of data per person and over 32,000 h of data over all participants. Other studies have exploited the size of data more by looking at the longitudinal data, and thus were able to apply different algorithms suited for large collections of samples [38].

### 4.1. Limitations

In our study, we used participants’ chronological age as an additional feature to improve our predictions. However, we only looked at people above the age of 65 years. We do not expect this method to be directly applicable to a younger population. As most younger adults are likely to have a cognitive ability around 30 in the tests used to measure cognitive ability in the presently used samples; a saturation effect is expected to kick in, making the usage of linear regression as a model no longer a good choice. Nevertheless, the usage of the reconstruction error might still be a valuable feature for other models.

In this study, measurements from 48 people were used. Due to the limited number of samples, a separation between people with mild cognitive impairment and those with severe impairment was not possible. For more in-depth analysis of the movement patters of the different health groups, more samples are necessary, specifically of people with severe cognitive impairments.

### 4.2. Future Work

The data we used in this study cover around four weeks of monitoring. The cognitive ability of people is not expected to change within this time frame. It would be interesting to assess whether longer monitoring periods could either improve the prediction of cognition, or alternatively be used to monitor change in the cognitive abilities.

In a newly established research facility, further studies regarding cognitive abilities, activities of daily living and home-based health assessments will continue [46].

## 5. Conclusions

The task in this paper was to evaluate indoor movement patters of elderly people and assess their usability as an indicator of cognitive abilities. This was performed using an analytical approach, where eigenvectors of the location matrix were calculated. The reconstruction error, obtained from the eigenvector decomposition, was then tested as a new feature for cognitive ability assessment.

Overall, the reconstruction error is a valid feature for the classification of the cognitive abilities into at least two groups, normal healthy people and those with mild to severe cognitive impairments.

The measurements necessary for obtaining this new feature are well accepted by the target group, as only contactless ambient sensors with a low level of privacy invasion are needed. Additional testing with larger groups, specifically those with severe cognitive impairments, would be needed for more insights into the precise behavioural aspects.

## Figures and Tables

**Table 1:** Partial correlation of cognitive-score, reconstruction error and age. * *p*-value <0.01; ** *p*-value <0.005.

	ρ
Age vs. Reconstruction error	0.27
Cognitive ability vs. Age	−0.38 *
Cognition score vs. Reconstruction error	−0.42 **

## Data Availability

Due to Swiss data regulations and the ethics committees, data can not be shared.

## Abbreviations

The following abbreviations are used in this manuscript:

MoCA	Montreal cognitive assessment
MCI	Mild cognitive impairment
AD	Alzheimer’s disease
MMSE	Mini-Mental State Examination
ADL	Activities of daily living
PIR	Passive infrared
ROC	Receiver operating characteristic
AUC	Area under the curve
RMSD	Root-mean-square deviation
